# Re-telling the story of aminoglycoside ototoxicity: tales from sub-Saharan Africa

**DOI:** 10.3389/fneur.2024.1412645

**Published:** 2024-06-28

**Authors:** Adebolajo A. Adeyemo, Babatunde Adedokun, Josephine Adeolu, Joshua O. Akinyemi, Olayemi O. Omotade, Odunayo M. Oluwatosin

**Affiliations:** ^1^Institute of Child Health, College of Medicine, University of Ibadan, Ibadan, Nigeria; ^2^Department of Otolaryngology, University College Hospital, Ibadan, Nigeria; ^3^Department of Epidemiology and Medical Statistics, College of Medicine, University of Ibadan, Ibadan, Nigeria; ^4^Department of Surgery, College of Medicine, University of Ibadan, Ibadan, Nigeria

**Keywords:** aminoglycosides, ototoxicity, streptomycin, hearing loss, tuberculosis, sub-Saharan Africa

## Abstract

**Background:**

Aminoglycosides, such as Streptomycin, are cheap, potent antibiotics widely used Sub-Saharan Africa. However, aminoglycosides are the commonest cause of ototoxicity. The limited prospective epidemiological studies on aminoglycoside ototoxicity from Sub-Saharan Africa motivated this study to provide epidemiological information on Streptomycin-induced ototoxicity, identify risk factors and predictors of ototoxicity.

**Method:**

A longitudinal study of 153 adults receiving Streptomycin-based anti-tuberculous drugs was done. All participants underwent extended frequency audiometry and had normal hearing thresholds at baseline. Hearing thresholds were assessed weekly for 2 months, then monthly for the subsequent 6 months. Ototoxicity was determined using the ASHA criteria. Descriptive statistics were used to analyze socio-demographic variables. Ototoxicity incidence rate was calculated, and Kaplan–Meier estimate used to determine cumulative probability of ototoxicity. Chi-square test was done to determine parameters associated with ototoxicity and Cox regression models were used to choose the predictors of ototoxicity.

**Results:**

Age of participants was 41.43 ± 12.66 years, with a male-to-female ratio of 1:0.6. Ototoxicity was found in 34.6% of the participants, giving an incidence of 17.26 per 1,000-person-week. The mean onset time to ototoxicity was 28.0 ± 0.47 weeks. By 28th week, risk of developing ototoxicity for respondents below 40 years of age was 0.29, and for those above 40 years was 0.77. At the end of the follow-up period, the overall probability of developing ototoxicity in the study population was 0.74. A significant difference in onset of ototoxicity was found between the age groups: the longest onset was seen in <40 years, followed by 40–49 years, and shortest onset in ≥50 years. Hazard of ototoxicity was significantly higher in participants aged ≥50 years compared to participants aged ≤40 years (HR = 3.76, 95% CI = 1.84–7.65). The probability of ototoxicity at 40 g, 60 g and 80 g cumulative dose of Streptomycin was 0.08, 0.43 and 2.34, respectively. Age and cumulative dose were significant predictors of ototoxicity.

**Conclusion:**

The mean onset time to Streptomycin-induced ototoxicity was 28 weeks after commencement of therapy. Age and cumulative dose can reliably predict the onset of Streptomycin-induced ototoxicity. Medium to long term monitoring of hearing is advised for patients on aminoglycoside therapy.

## 1 Introduction

The most common disability globally is hearing loss, 432 million adults and 34 million children have disabling hearing loss (1), and within the next two decades people with disabling hearing loss will increase to more than 900 million (1). The etiology of hearing loss is multiple; however, many are preventable, including drug-induced ototoxicity (2, 3).

Aminoglycosides such as Streptomycin, Kanamycin, and Amikacin are powerful antibiotics but known to elicit harmful side-effects such as ototoxicity. Aminoglycoside-induced ototoxicity arising from injury to the cochlea sensory hair cells and stria vascularis are thought to be permanent (3, 4), though neural degeneration without concomitant damage to the cochlear hair cell can also occur (5). This dose-dependent adverse effect (6) is worsened by the narrow therapeutic range of aminoglycosides and the inconsistencies in the drug pharmacokinetics in different persons (7).

Aminoglycoside-induced ototoxicity begins initially in the high frequency sound range before progressing to involve the lower frequencies sounds (including speech frequencies) (8). This pathognomonic feature of aminoglycoside-induced ototoxicity is adduced to hair cell damage that starts initially at the cochlea basal turn, before gradual advancement to involve hair cells at the cochlea apex (9). The arrangement of the hair cells within the human cochlea places hair cells sensitive to high frequency sounds at the base while hair cells sensitive to low frequency sounds are near the apex, this arrangement explains further the classic presentation of aminoglycoside-induced ototoxicity.

There is a wide variation in the incidence of aminoglycoside ototoxicity reported in the literature from 28 to 37% (10, 11), this is probably due to variations in the benchmarks of evaluation for hearing loss, patient classification and treatment guidelines in the various studies (12). Application of more sensitive testing standards increases the incidence of ototoxicity to 47% (13, 14). The incidence of ototoxicity also depends on the drug dosage and length of administration, thus when treatment is prolonged for up to 6–12 months the incidence of ototoxicity is up to 100% (12, 15).

Mankind had been infected with *Mycobacterium tuberculosis* since antiquity, yet the disease still has a large global footprint, with a third of the human population already infected with *Mycobacterium tuberculosis* and are liable to succumb to a full-blown disease (7, 16). TB accounts for over two million mortalities every year, and nine million individuals are newly diagnosed yearly (17). The previous World Health Organization (WHO) recommendation was a Tuberculosis (TB) re-treatment regimen that included 2 months of daily Streptomycin injection. The key handicap in long-term administration of parenteral aminoglycosides is toxicity.

Despite the well-known side effects of aminoglycosides, there is massive prescription and administration of these drugs in many low- and medium-income countries. The cheap price of the drugs due to the low manufacturing costs (12) coupled with lax regulatory oversight makes aminoglycoside antibiotics popular in societies with limited purchasing power. Home storage coupled with self-medication is also seen with aminoglycosides despite the parenteral route of administration (18). A likely scenario is that aminoglycoside-induced ototoxicity could significantly contribute to the prevalence of hearing loss in these societies. Moreover, despite the various documentations of aminoglycoside ototoxicity, the critical cut-off dose among Nigerians and Sub-Saharan Africans is not known, even though there is wide consumption of the drugs on the continent. Moreover, the relationship of demographic parameters with aminoglycoside ototoxicity is not fully known. The sparse data about the toxicity that could ensue following the massive use of aminoglycosides in the population motivated this study. The goal of this study is to provide data on aminoglycoside-induced ototoxicity in a patient cohort. Thus, this study determined the incidence of ototoxicity, the probability of ototoxicity occurrence, the median ototoxicity onset time, the critical cumulative cut-off dosage and the demographic and pharmacologic parameters associated with aminoglycoside-induced ototoxicity in a cohort of patients being re-treated following an initial curative failure of TB.

## 2 Materials and methods

This was a study of ototoxicity among a cohort of individuals diagnosed with pulmonary TB an who underwent treatment with streptomycin based anti-TB drugs. Subjects were recruited successively from Directly Observed Therapy-Short course (DOTS) centers in Ibadan, Southwest Nigeria as diagnosis was made and informed consent provided. DOTS centers are embedded in hospitals, clinics or primary health care centers, streptomycin injections were administered to the study population by the health care workers in the DOTS centers. Ethical approval was granted by the Oyo State Ministry of Health, Ethical Review Committee (AD 13/479).

### 2.1 Study population

TB patients (≥18 years) with physician diagnosed TB undergoing second line of treatment with anti-TB drugs which included Streptomycin, Rifampicin, Isoniazid, Pyrazinamide, and Ethambutol. These patients had previously been treated for TB and declared cured or completed a full course of treatment and once again developed sputum smear-positive TB or a sputum smear positive patient who while on treatment remains or became smear positive again 5 months or more after commencement of treatment. Other eligibility criteria were hearing thresholds within the normal range as assessed by pure tone audiometry (PTA) at baseline before commencement of the second line of treatment. The following cadres of patients were excluded: those with previous history of hearing loss secondary to any pathology and/or signs of ear infection after being examined, presence of hearing loss as assessed by PTA at baseline**, a**dditional usage of medications with ototoxic potentials and/or nephrotoxic potentials and concurrent HIV/AIDS infection. Using the minimum sample size calculation for cohort studies (19), the sample size estimate was 138 subjects.

### 2.2 Data collection

All relevant socio-demographic and medical information were documented with the aid of a modified questionnaire (20) which was administered by an interviewer. Supplemental medical data was extracted from hospital folders of the patients. Baseline pre-treatment pure tone audiometry with a KUDU wave audiometer (Geoaxon, Pretoria, South Africa) was done for every enrolled subject between 125 Hz and 8,000 Hz (10), subsequently the pure tone audiometry was performed again every week between 125 Hz and 16,000 Hz throughout the first 2 months of the therapy. After that the pure tone audiometry was performed for all enrolled subjects every month for the following 6 months of the therapy. At every visit with the study subjects, questions were asked on subjective hearing changes. Serum streptomycin levels was not measured during the course of therapy (10). The guidelines of the National Tuberculosis and Leprosy Control Program mandates single intramuscular Streptomycin injection (either 0.5 g, 0.75 g or 1 g depending on the weight) daily for patients undergoing TB re-treatment thus single daily dosing regimen was adopted for this study. All the drugs used in TB management are supplied centrally by the National Tuberculosis and Leprosy Control Program, the Program guidelines did not stipulate refrigeration of drugs.

The American Speech-Language-Hearing Association guidelines were utilized in computing ototoxic threshold shift (sensorineural hearing loss) from the baseline audiogram: (1) a reduction of 20 dB or more in any specific test frequency, (2) a reduction of 10 dB or more at any two adjacent frequencies, or (3) loss of response at three consecutive frequencies where responses were previously obtained (21).

Grading the severity of the ototoxicity with the NCI-CTCAE (National Cancer Institute Common Terminology Criteria for Adverse Events) version 5 was done. This system of grading is one of the most widely cited classification of ototoxicity, it rates severity of ototoxicity on a scale of 1–4, based on changes in the audiometric thresholds, number of frequencies affected, and indication for intervention (22). Grade 1 is denoted as a mild adverse event and intervention is not indicated, while grade 2 is denoted as moderate adverse event warranting minimal intervention, grade 3 is severe adverse event; and grade 4 indicates the most severe ototoxicity grade requiring urgent intervention (23). NCI-CTCAE, like several ototoxicity monitoring protocols rely on differences in hearing threshold from a baseline audiogram. The subjects who gave history of hearing loss or who had audiograms suggesting hearing loss were appropriately counseled and given referrals to see an Otolaryngologist.

### 2.3 Statistical analysis

The cumulative dosage of Streptomycin received by each participant was determined. The incidence of ototoxicity was analyzed using demographic procedure of computing incidence rate. The Kaplan–Meier estimate was used to determine probability of developing ototoxicity and Cox regression models were used to choose the predictors of ototoxicity.

## 3 Results

The number of recruited subjects was 153 adults, there were 95 males and a gender ratio of M: F, 1: 0.6. The 40–49 years age group and the <40 years age group had greater representation among the participants (32 and 44% respectively). Majority of the participants had only primary education (37.9%) and were married (78.4%). Most of the participants were either laborer’s or petty traders (62.4%) and were of the Yoruba tribe (97.4%). The constituents of the socio-demographic parameters of the study subjects are outlined below (Table 1).

**Table 1:** Socio-demographic characteristics of study subjects.

Variable	Frequency (*N* = 153)	Percentage (%)
Age (years)		
< 40	68	44.4
40–49	49	32.0
50+	36	23.5
Gender		
Male	95	62.1
Female	58	37.9
Education		
No formal education	8	5.2
Primary	58	37.9
Junior secondary	53	34.6
Senior secondary	20	13.1
Tertiary	14	9.2
Marital status		
Married	120	78.4
Single	26	17.0
Divorced	4	2.6
Missing	3	2.0
Occupation		
Professionals/ Civil servants	10	5.8
Drivers/artisans	35	20.6
Petty traders/laborer’s	106	62.4
Unemployed, fulltime housewife	19	11.2
Tribe		
Yoruba	149	97.4
Ibo	2	1.3
Hausa	2	1.3

Knowledge of signs of TB was high among the participants, 92.2% of the participants were aware of signs of TB. However, in contrast only a tenth of the participants could ascertain how they contracted the disease. The proportion of participants who were aware of possible side effects of TB treatment on the ear was 7.8%, only 2% had ever had any form of hearing assessment, and 32% had experienced some form of hearing-related symptoms. Majority of the participants (63.4%) received <0.75-gram dosage of Streptomycin while 32% received 1gram dosage of Streptomycin. The study participants were nearly evenly distributed in the three weight categories (Table 2).

**Table 2:** Distribution of weight and dosage.

Variable	Frequency	%
Weight at presentation (kg)		
<50	49	32.0
50–59	60	39.2
60 and above	43	28.1
Dose of streptomycin (gram)		
≤0.75	104	68.0
1	49	32.0

### 3.1 Prevalence of ototoxicity

**Figure 1:**
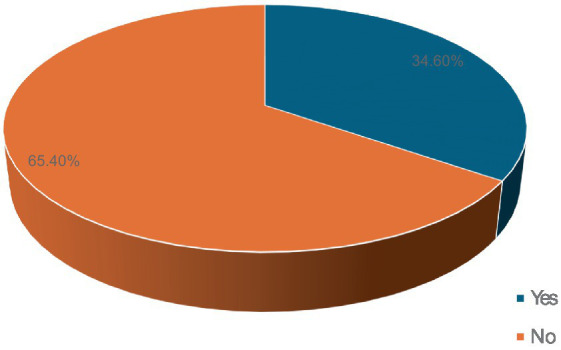
Proportion of respondents with ototoxicity.

**Figure 2:**
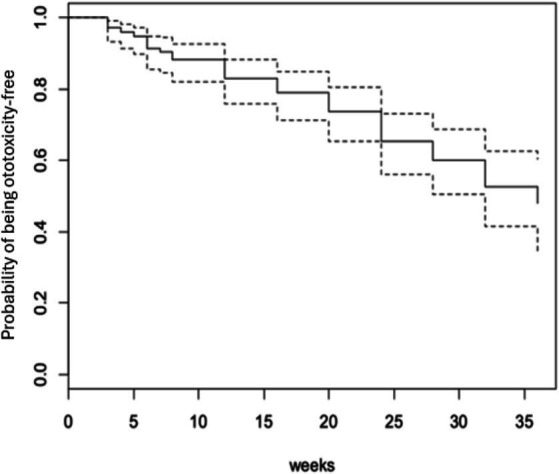
Kaplan Meier estimate curve of the overall time to ototoxicity of TB patients.

Fifty-three participants (34.6%) developed ototoxicity (Figure 1) corresponding to an incidence of 17.26 per 1,000-person weeks of follow up. The median time before ototoxicity onset being 24 weeks (range = 33 weeks). However, the Kaplan Meier estimate of the mean time to development of ototoxicity was 27.8 weeks (95% CI = 25.9–29.7; Figure 2). Figure 2 also shows the cumulative proportions of participants with ototoxicity at different follow up time points. Almost 85% were free of ototoxicity by the 10th week, and about 75% by the 20th week. By the 30th week about 60% had no ototoxicity.

The severity grade of ototoxicity (Common Terminology Criteria for Adverse Events) showed that Grade 1 and Grade 4 had the highest frequency of 21 (39.6%) while Grades 2 and 3 had 7.5 and 13.2%, respectively.

### 3.2 Probability of ototoxicity and factors associated with ototoxicity

**Figure 3:**
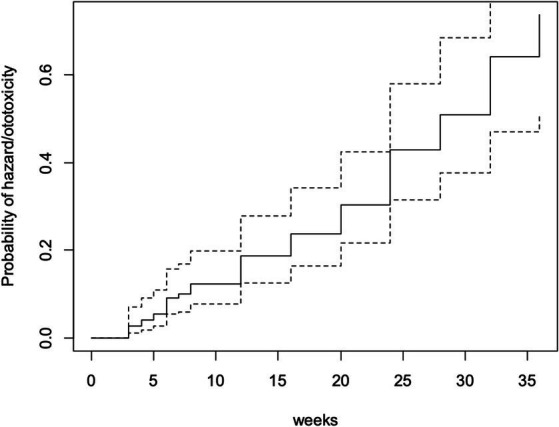
Cumulative hazard function.

By the 28th week of follow-up (which was the mean ototoxicity-free time), hazard rate for developing ototoxicity was 0.507, i.e., the risk or probability of a respondent for developing ototoxicity having survived (i.e., remaining free of ototoxicity) to 28 weeks. At follow-up completion (36 weeks), the hazard rate of respondents was 0.736 (Figure 3).

**Figure 4:**
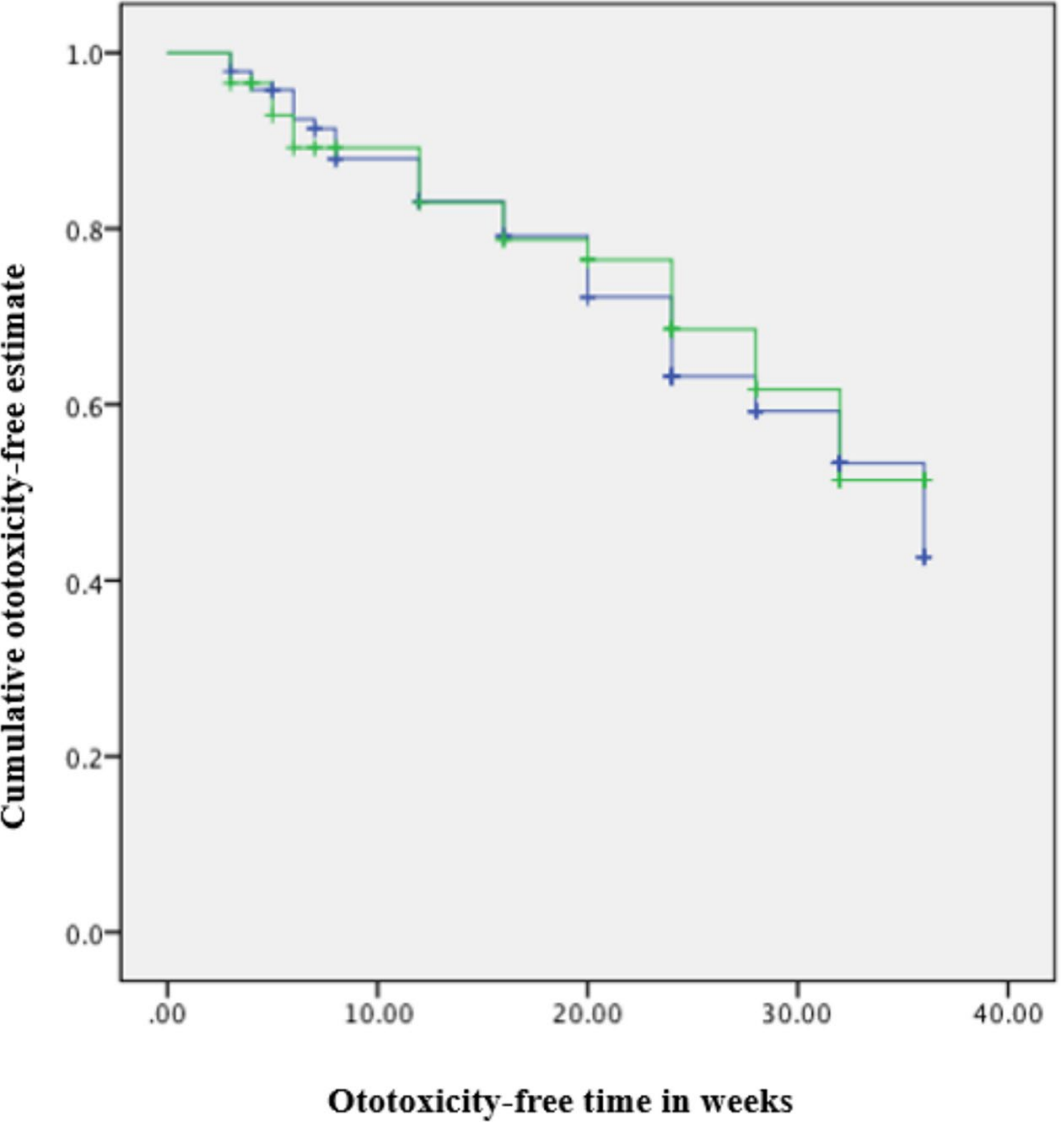
Kaplan–Meier estimate curve: ototoxicity onset by gender (censored denotes individuals with ototoxicity). Green line: Female. Blue line: Male.

A significant difference in time to ototoxicity was found between age groups, with longest times to ototoxicity found among younger participants aged less than 40 years, followed by those aged 40–49 years, and lowest among those 50 years and above (*p* < 0.001; Table 3). No significant association was found between time to ototoxicity and gender (*p* = 0.764), weight (*p* = 0.562), dose (*p* = 0.486), and previous ear symptoms (*p* = 0.304). The Kaplan Meier plot of cumulative ototoxicity-free time by gender showed that the two plots of ototoxicity-free times seem to overlap though as from around the 20th week of follow up the cumulative ototoxicity-free time of females is higher than that of males but is lower after the 30th week (Figure 4).

**Table 3:** Comparison of factors influencing time to ototoxicity.

Variable	Number of events (*n* = 53)	Mean time to ototoxicity in weeks (SD)	Median time to ototoxicity in weeks (range)	Log rank Chi square	*p* value
Gender				0.09	0.764
Male	34	20.1(10.1)	24(33)		
Female	19	20.0(10.8)	22(33)		
Age				15.33	<0.001
<40	14	21.2(11.0)	24(33)		
40–49	18	19.7(10.4)	20(33)		
50+	21	18.3(8.9)	18(33)		
Weight				0.34	0.562
<50	20	21.5(9.2)	24(32)		
50–59	17	22.2(10.2)	24(33)		
60+	16	15.5(10.5)	12(33)		
Previous ear symptoms				1.06	0.304
Yes	21	18.8(10.2)	20(33)		
No	32	20.8(10.4)	24(33)		

**Figure 5:**
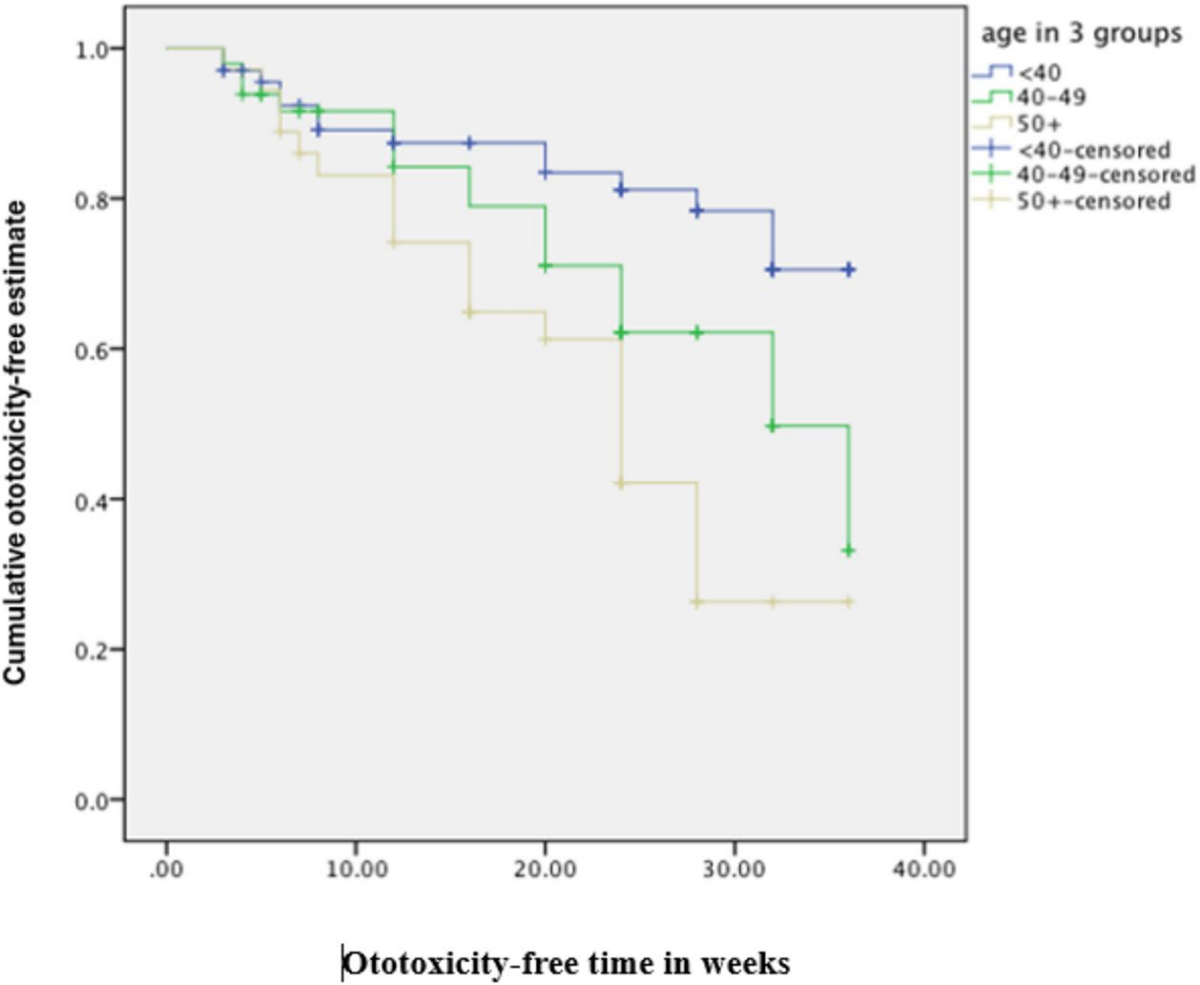
Kaplan–Meier estimate curve comparing ototoxicity onset by age group (censored denotes individuals with ototoxicity).

**Figure 6:**
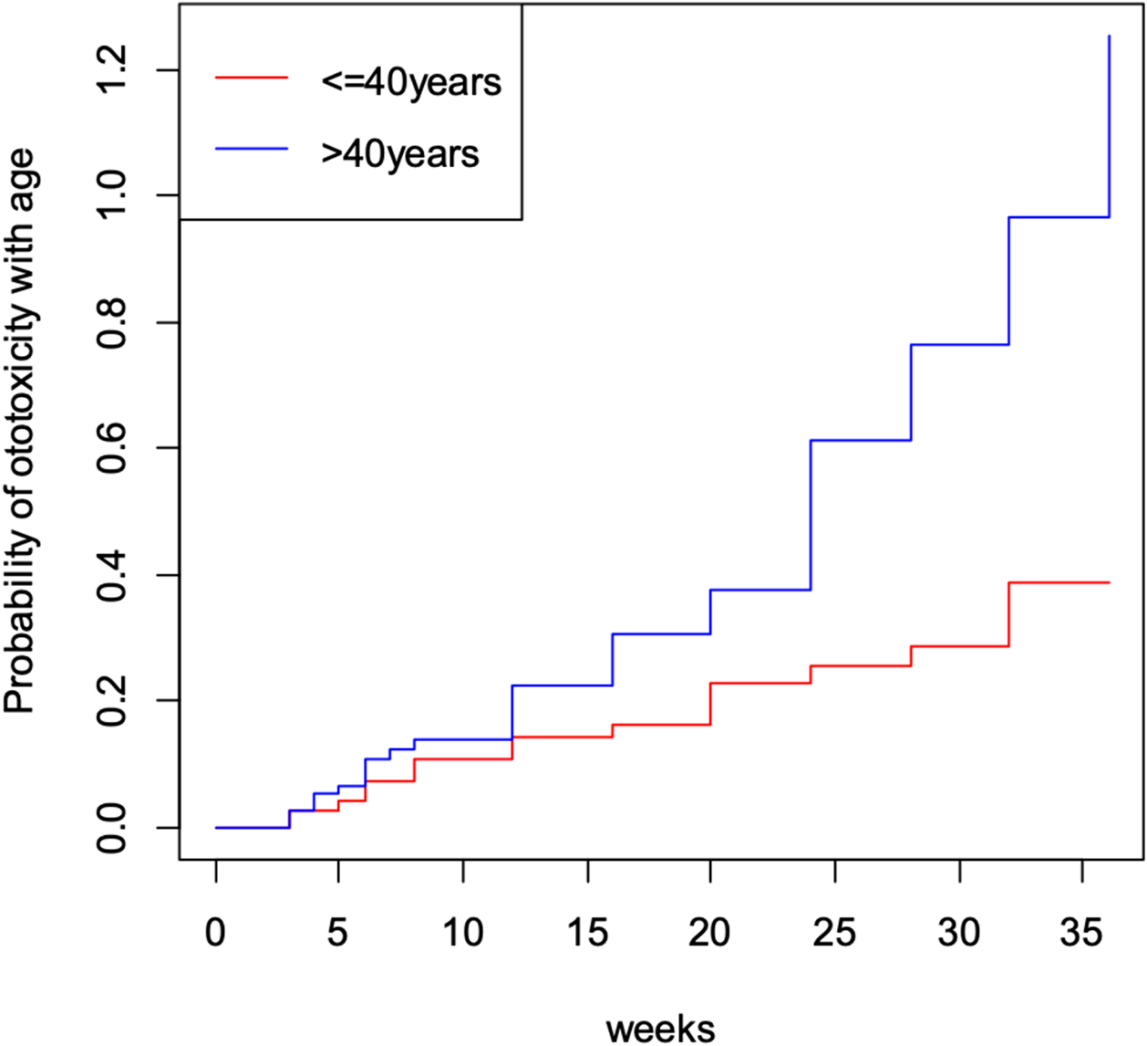
Kaplan–Meier estimate curve showing probability of ototoxicity by age.

**Figure 7:**
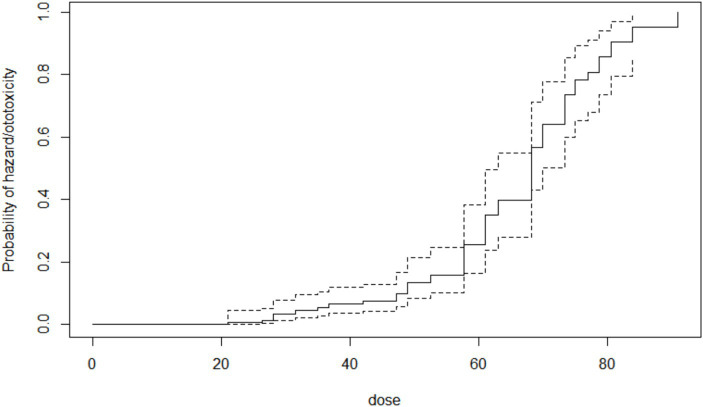
Kaplan–Meier estimate curve showing probability of ototoxicity by cumulative dose x-axis is the cumulative dose of Streptomycin received.

The cumulative time to ototoxicity was consistently higher among younger participants aged less than 40 years indicating longer times to experiencing ototoxicity compared to older participants. The difference was most marked as from 25 weeks after follow-up (Figure 5). A statistically significant difference was observed (*p* < 0.001; Table 3). Comparing the risk of developing ototoxicity after re-classifying the ages into two age groups, it was seen that by 28th week, the risk of developing ototoxicity for respondents below 40 years of age was 0.288 while it was 0.766 for those older than 40 years (*p* value = 0.004; Figure 6). Comparison of the probability of ototoxicity with cumulative dosage showed that the probability of developing ototoxicity increased with increasing cumulative dosage of Streptomycin (Figure 7).

The crude, unadjusted hazard ratios from Cox regression of time to ototoxicity showed that participants aged 50 years and above were 3.6 times more likely to have ototoxicity (95% CI = 1.81–7.16) compared to participants aged 30–39 years. Those in the age group 40–49 years were twice more likely to have ototoxicity, but this higher hazard was not statistically significant (95% CI = 0.99–4.02; Table 4). Participants weighing 50–59 kg were significantly twice less likely to have ototoxicity compared with participants weighing 60 kg and above (95% CI = 0.25–0.99; Table 4). Multiple cox regression analysis of time to ototoxicity on variables showed that the hazard of hearing impairment was significantly higher among those aged 50 years (HR = 3.76, 95% CI = 1.84–7.65) when likened to participants aged 40 years and younger. No significant differences were observed in hazards of ototoxicity between other characteristics (Table 5).

**Table 4:** Crude hazard ratios from Cox regression of ototoxicity onset on socio-demographic characteristics and past symptoms.

Variable	Crude hazard ratio (CHR)	95% confidence interval CHR	*p*-value
Age (years)			
30–39 (ref)	1	0.99–4.02	0.053
40–49	2.00	**1.81–7.16**	**<0.001**
50+	3.60		
Gender			
Male	1.09	0.62–1.91	0.770
Female (ref)	1		
Weight (kg)			
Less than 50	0.77	0.40–1.48	0.427
50–59	0.50	**0.25–0.99**	**0.049**
60+ (ref)	1		
Previous ear symptoms			
Yes	1.32	0.76–2.30	0.319
No	1		

**Table 5:** Adjusted hazard ratios from Cox regression of ototoxicity onset on socio-demographic characteristics and past symptoms.

Variable	Adjusted hazard ratio (AHR)	95% confidence interval AHR	*p*-value
Age (years)			
30–39 (ref)	1	0.99–4.08	0.052
40–49	2.01	1.84–7.65	<0.001
50+	3.76		
Gender			
Male	1.23	0.69–2.20	0.478
Female (ref)	1		
Weight (kg)			
Less than 50	1.13	0.49–2.58	0.777
50–59	0.60	027–1.33	0.206
60+ (ref)	1		
Previous ear symptoms			
Yes	1.25	0.70–2.21	0.457
No	1		

## 4 Discussion

### 4.1 Factors associated with TB susceptibility

The results of this study showed predominance of male patients, similar to other studies which showed male preponderance among patients with pulmonary TB (24–28). The average male/female ratio of TB worldwide is 1.9 ± 0.6, though male/female ratio values as high as 3.0 has been recorded in some countries (29). The male preponderance of pulmonary TB is clearly chronicled in every territory worldwide (30–35). There is a bi-directional relationship between TB and poverty without a clear starting point. Most of the study subjects (77.7%) had 9 years or less of accumulated formal education. This data demonstrates the skewed relationship between nominal education and TB infection (36–38). Indeed, it is known that the extent of the educational inequality associated with TB is far greater than what is seen in other diseases (34).

### 4.2 Incidence of aminoglycoside-induced ototoxicity

The study revealed an incidence of 34.6% for aminoglycoside-induced ototoxicity. Incidence of ototoxicity is commonly understated, probably due to the initial sparing of frequencies within the speech range in the early phase of aminoglycoside-induced ototoxicity. The lack of interference with daily communication implies that patients often fail to notice the adverse effect and thus do not report it. Moreover, reports from clinical studies may be hampered by criteria used in defining ototoxicity, differences in methods of hearing assessment and inadequate duration of hearing threshold monitoring to aid identification of lesions occurring very late after cessation of drug therapy (39).

This study’s incidence rate is comparable to previous studies of aminoglycoside ototoxicity (10), and within the range of 17 to 47% documented earlier (40, 41). About a third of the patients in the cohort developed ototoxicity clearly demonstrating the negative impact of aminoglycosides usage on the hearing profiles of individuals and cementing what is already known about the adverse effect of aminoglycoside antibiotics.

Further evaluation was done by grading the severity of the ototoxicity with the NCI-CTCAE (National Cancer Institute Common Terminology Criteria for Adverse Events) version 5. Grading ototoxicity severity may influence clinical decisions on continuity of therapy (42), and are essential for comparison of results from different clinical sites through application of uniform guidelines, however, there is paucity of publications on the grading of severity of aminoglycoside ototoxicity. The NCI-CTCAE grading system however has certain limitations: it discountenances ultra-high frequencies, even though hearing loss in ototoxicity begins at these ultra-high frequencies (43), the middle grades of NCI-CTCAE (grades 2 and 3) are also said to be imprecise; the hearing loss in these grades is defined as spanning between 25 and 80 dB loss; this loss of precision may hinder rendering the consequence of the grades to daily life activities of a patient (43). Despite these observed shortcomings the grading system remains widely cited.

A very significant proportion of the patients had Grade 4 severity—the most severe form of the adverse event. This may have occurred due to the long follow up period in the study, highlighting the progressive long-term effects of aminoglycoside ototoxicity. Aminoglycosides amass within the cochlea and persists in inner ear tissues for long periods after clearance from the bloodstream, accounting for delayed death of cochlear hair cells seen after termination of treatment (44). The significant Grade 4 proportion also demonstrates the burden of aminoglycoside ototoxicity in the society, especially in countries with high consumption rates (8, 45–48).

### 4.3 Ototoxicity development timeline

The mean onset time of aminoglycoside ototoxicity was 27.8 weeks in this study. A much shorter mean onset time of 9 weeks was recorded among heterozygous cohort of patients with drug resistant TB on Kanamycin therapy (49), as well as individuals with other clinical conditions such as HIV infection, diabetes mellitus and hypertension. These co-morbidities are independently related with hearing loss and the drug regimens for these co-morbidities could also adversely affect hearing (50–52), hence the much shorter onset time of ototoxicity (53). A key importance of ascertaining the onset of ototoxicity is utilization in planning ototoxicity monitoring programs. Early identification of ototoxicity can avert or reduce hearing loss that could reduce quality of life following treatment. It is imperative that when considering implementation of ototoxicity monitoring program to consider initiation of the monitoring at least 6 weeks after exposure to aminoglycosides in patients without additional co-morbidities and much earlier in patients with additional co-morbidities.

### 4.4 Factors influencing onset of ototoxicity

Age of the subjects was significantly associated with onset of ototoxicity; younger people are least likely to develop ototoxicity. The relationship between advancing age with the onset of aminoglycoside ototoxicity has been demonstrated in other studies also (11, 49). The impact of the cumulative insult on the inner ear from various assaults such as noise, and the aging process could account for the worse outcome of ototoxicity seen in older age groups.

Chronic inflammation—also known as “inflammaging”—is a pervasive feature in all tissues as the body ages (54–57). The aging cochlea exists in such a chronic inflammatory state, in addition, decline in mitochondrial function with subsequent reduction in energy production (58), atrophy of the stria vascularis and ensuing disruption in blood supply (59) are some of the possible mechanisms by which increase in age worsens cochlea function including cochlea response to external insults such as aminoglycoside ototoxicity.

Damaging the cochlear mitochondria is key step in the pathogenesis of aminoglycoside induced ototoxicity (60). The cochlea organ has steep energy requirements to sustain the endo-cochlear potential generated by the stria vascularis, thus there is high concentration of mitochondria within the different cellular components of the cochlea for energy production through cellular respiration (61). The high energy stipulation for optimal function of the cochlear makes the organ susceptible to the aging process. Thus, the insidious ototoxicity effect of aminoglycosides is worsened by the aging process, this explains further the association between increase in age and ototoxicity onset (62, 63). Significant necroptosis and proinflammatory responses are seen in the aging cochlea (62), this may have a synergistic effect with the ototoxic potential of aminoglycosides and thus contributes to the association between age and onset of ototoxicity.

A statistically significant relationship was not observed between gender and ototoxicity onset in this cohort. Though there were more males who developed ototoxicity than females within the cohort. The predominance of male subjects with ototoxicity was probably not directly related to the gender variation of the cohort. The M:F ratio in the cohort is 1.6:1, however, the M:F ratio among those with ototoxicity was 1.8:1; suggesting that there was a greater incidence of ototoxicity in the male gender despite the gender variation in the main cohort. This is in keeping with previous reports of hearing loss being more common in males (64). However, other authors found a different observation, gendered differences were seen in response to drugs—in pharmacodynamics and pharmacokinetics—manifesting as increased disposition of females to drug ototoxicity among other adverse drug conditions (65, 66). Similarly, in a cohort of patients with nontuberculous mycobacteria pulmonary disease who received an aminoglycoside therapy—amikacin—females in the cohort had greater vulnerability to initiation of ototoxicity compared to males (67).

No statistically significant association was observed between subjects’ weight and ototoxicity development. Even though a causal relationship between weight and hearing loss has not been established (68), a relationship between both conditions is plausible (68–78). It is possible that the use of weight alone as an indicator of the BMI—as used in this study—may be insufficient to accurately predict relationship with onset of ototoxicity. Moreover, the weight of the subjects may not be taken in isolation for predicting ototoxicity onset, there could be interplay of several modulators such as adipose tissue distribution, gender and age that could interconnected functions in determining the onset of ototoxicity.

The study showed that increasing the cumulative dose of Streptomycin was associated with a significant increase in the likelihood of ototoxicity (10, 79). The presence of aminoglycosides in the cochlea 11 months post completion of therapy (80) suggests reduced clearance from the cochlea and this may be a possible contributor to aminoglycoside ototoxicity (81). Cumulative dose may thus indicate the degree of accumulation of the aminoglycosides in the inner ear, and a pointer to the possible degree of toxicity. A very sharp increase in the probability of ototoxicity was seen when the cumulative dosage reached 70 g among subjects in this study, other studies have also shown that every 10-fold increment in the cumulative dose of aminoglycoside increased the odds of ototoxicity by 6.9-fold (10). This dose-dependent nature of aminoglycoside ototoxicity with its narrow therapeutic window, suggests the need for strict regulation of the drug usage, particularly for patients on hospital admission (79) and/or when the drug is to be used for extended periods. Thus, it is advocated to offer therapeutic drug monitoring in the treatment of TB patients on aminoglycosides with the goal of reducing cumulative dosage and ototoxicity while maintaining efficacy, instead of the current WHO recommendation of dosing based on body weight, resulting in lower dosing based on serum concentrations of the drug (82). This approach of using pharmacokinetic/pharmacodynamic parameters has been proven to be a driver of successful therapy (83). However, this personalized approach is fraught with multiple challenges in low resource settings. Adopting this precision medicine approach will require provision of serum aminoglycosides levels in high-throughput rates for swift and precise results, as well as evaluating susceptibility to drug to determine precise MIC values (84), this implies provision of trained and experienced personnel and appropriate equipment. Surmounting the cost barriers for a national roll-out of this personalized care may be impractical in the short term in many low- and medium-income countries, however, centralizing analytical facilities to concentrate knowledge/personnel and reduce costs may be a feasible option (82).

## 5 Limitations

This study had certain limitations, the National Tuberculosis and Leprosy Control Program in Nigeria guidelines does not include monitoring of serum aminoglycosides and serum creatinine monitoring levels in the patients undergoing TB treatment thus these were not done in this study.

## 6 Conclusion

This study filled a critical gap by providing longitudinal data on the incidence of aminoglycoside ototoxicity after extended monitoring of study subjects, including the prevalence of ototoxicity in a cohort of individuals on long term aminoglycoside therapy, identification of risk factors and predictors of ototoxicity. The mean time to onset of Streptomycin-induced ototoxicity was determined to be 28 weeks after initiation of therapy. This may be useful in designing screening programs for hearing loss in patients receiving aminoglycoside therapy. Knowing when to expect the onset of ototoxicity will assist in the proper allocation of resources to ensure maximum yield while eliminating unnecessary redundancy in health care.

In addition, it is appropriate to counsel patients receiving aminoglycoside therapy not only about the risk of ototoxicity, but also the possibility of late onset of ototoxicity after initiation of therapy, and the need for active hearing conservation efforts. Medium- to long-term monitoring of hearing should also be provided for these patients. Personal monitoring of hearing by patients receiving or completing aminoglycoside therapy should be encouraged. These individuals can use free mobile applications such as the “hearWHO” app developed by the World Health Organization (85). This will allow early detection of hearing loss and appropriate interventions.

## Data availability statement

The original contributions presented in the study are included in the article/supplementary material, further inquiries can be directed to the corresponding author.

## Ethics statement

The studies involving humans were approved by Oyo State Ministry of Health, Ethical Review Committee. The studies were conducted in accordance with the local legislation and institutional requirements. The participants provided their written informed consent to participate in this study.

## Author contributions

AA: Writing – review & editing, Writing – original draft, Resources, Project administration, Methodology, Investigation, Funding acquisition, Data curation, Conceptualization. BA: Writing – review & editing, Formal analysis, Data curation. JA: Writing – review & editing, Formal analysis. JOA: Writing – review & editing, Formal analysis. OOO: Writing – review & editing, Supervision. OMO: Writing – review & editing, Validation, Supervision.
